# A Maternal Influence on Reading the Mind in the Eyes Mediated by Executive Function: Differential Parental Influences on Full and Half-Siblings

**DOI:** 10.1371/journal.pone.0023236

**Published:** 2011-08-05

**Authors:** Gillian Ragsdale, Robert A. Foley

**Affiliations:** Leverhulme Centre for Human Evolutionary Studies, University of Cambridge, Cambridge, United Kingdom; Kyushu University, Japan

## Abstract

**Background:**

Parent-of-origin effects have been found to influence the mammalian brain and cognition and have been specifically implicated in the development of human social cognition and theory of mind. The experimental design in this study was developed to detect parent-of-origin effects on theory of mind, as measured by the ‘Reading the mind in the eyes’ (Eyes) task. Eyes scores were also entered into a principal components analysis with measures of empathy, social skills and executive function, in order to determine what aspect of theory of mind Eyes is measuring.

**Methodology/Principal Findings:**

Maternal and paternal influences on Eyes scores were compared using correlations between pairs of full (70 pairs), maternal (25 pairs) and paternal siblings (15 pairs). Structural equation modelling supported a maternal influence on Eyes scores over the normal range but not low-scoring outliers, and also a sex-specific influence on males acting to decrease male Eyes scores. It was not possible to differentiate between genetic and environmental influences in this particular sample because maternal siblings tended to be raised together while paternal siblings were raised apart. The principal components analysis found Eyes was associated with measures of executive function, principally behavioural inhibition and attention, rather than empathy or social skills.

**Conclusions/Significance:**

In conclusion, the results suggest a maternal influence on Eye scores in the normal range and a sex-specific influence acting to reduce scores in males. This influence may act via aspects of executive function such as behavioural inhibition and attention. There may be different influences acting to produce the lowest Eyes scores which implies that the heratibility and/or maternal influence on poor theory of mind skills may be qualitatively different to the influence on the normal range.

## Introduction

This study focuses on the test, ‘Reading the mind in the eyes’ (henceforth referred to as Eyes), and has two related parts. Firstly, full and half-sibling pairs' Eyes scores were analyzed to detect differential parental influences. To further determine the character of any influence, the second part of the analysis assesses how Eyes scores relate to theory of mind (ToM), empathy and executive function. Our aim here is to consider what cognitive function Eyes is measuring.

Eyes requires subjects to choose the most accurate of four descriptions for the expression in each of 36 pairs of eyes, on the premise that this ability is a measure of theory of mind, i.e. the ability to infer another person's mental state [1]. The ability to read or interpret facial expressions has been associated with measures of empathy and/or ToM and impairment of this skill is associated with impaired social competence such as that seen in autism spectrum condition (ASC) [2]. The eyes were chosen, rather than the whole face, as adults have been found to read complex mental states (rather than basic emotions) equally well from the eyes alone as from the whole face, and adults with ASC were especially poor at interpreting the eyes alone [3].

### Reading facial expression and ToM

Research into the relationship between reading facial expression and ToM is dominated by the study of pathologies in which individuals have deficits in one or both abilities, such as ASC, Turner's Syndrome and schizophrenia. Individuals with ASC and those with Turner's Syndrome fail to recognize facial expressions of fear or ascertain gaze direction, both components of ToM [4] [5].

Sabbagh and Seamans [6] found that parents' Eyes scores were positively correlated with false-belief style measures of ToM applied to their 3–4 year-old children. This not only implies, somewhat unsurprisingly, that there are some sort of parental influences on ToM (whether genetic or environmental) but more usefully that Eyes does actually relate directly to ToM in the *normal population* as measured by the ability to understand false beliefs. False belief tests, which test the ability to understand that someone can hold a different belief (such as that a toy which has been moved is still in the place it was) have generally been the gold standard for measuring ToM but these kinds of tests tend to reach a ceiling when administered to normally functioning adults [7] Also, eye gaze processing has been found to be correlated with ToM in developing children and adolescents i.e. inferring the locus of attention of a face correlated with the ability to attribute intention to cartoon shapes [8].

### ToM, empathy and executive function

Empathy is increasingly taken to comprise at least two distinguishable components: emotional and cognitive empathy, the latter frequently taken as synonymous with ToM [9] [10] [11]. Executive function comprises higher cognitive functions such as verbal fluency, attention switching, behavioural flexibility, planning and organization [12].

The literature on ToM and its relationship to executive function is inconsistent and sometimes contradictory: a recent review of 24 studies concluded that executive function and ToM were ‘tightly associated’ but could not consistently identify subcomponents of executive function to be associated with ToM [13]. This inconsistency appears to stem largely from the diverse range of measures used, especially for ToM. Other commonly used tests include Strange Stories [14], Faux Pax [15], reading full facial expressions [16] and the Eyes test used in this present study. In some cases these tests, being regarded as genuine measures of ToM, are consolidated as one global score [17]. The relationship between these tests though, is far from clear. Eyes has been found to correlate strongly with Faux Pas scores: r = 0.428, p = 0.01 [17] but Ahmed et al. [18], found no correlation between Eyes, Strange Stories sand Faux Pas scores or consistent relationships with a range of executive function scores in a sample of 135 adults from Georgia University. Differences between these measures supports the view that there are multiple domains to ToM which are supported by different components of executive function [19]. Bull et al. [20] carried out a dual task study using an adapted form of Eyes, another more complicated ToM test and executive function tasks and found Eyes to depend on executive function, principally inhibition rather than attention.

Evidence that ToM and executive function use the same neural processes comes from the co-morbidity of deficits in ToM and executive function found in some children with brain injuries [21] and neurological studies of normal children and adults [22] [23]. However, cases where adults have a deficit in either executive function or ToM but not both do suggest that there is some degree of independence between the two [24]. There is also evidence that the relationship between executive function and ToM changes of over time, with considerable interdependence in infancy and childhood which decreases, or even ceases in adulthood [25].

Evidence for ToM, as mediated specifically by facial expression recognition, having independent, domain-specific development comes from neuroimaging studies of specific kinds of expression. The recognition and processing of some specific emotions is associated with specific neural pathways (e.g. fear and the amygdala: [26]; disgust and the insula: [27] [28]. This supports the notion that the processing of specific emotions have independent evolutionary histories, reflecting different selective pressures [29] and consequently there is no central processing area in the brain for emotions in general. Areas of the brain associated with processing facial expressions in some cases overlap with areas associated with executive function tasks but may also be independent [24] [30] Mirror neurons in the inferior frontal gyrus [31] are also thought to facilitate ToM by employing the same neural substrates as imitation [32] which sends information to limbic areas such as the amygdala via the insula in order to produce the emotional outcome [33].

### Genetic and environmental influences on ToM

There is evidence for both genetic and environmental influences on empathy and ToM. However, research varies considerably concerning the amount of variance in individual differences in ToM attributable to genetic, shared environmental and non-shared environmental influences. Hughes and Cutting [34] attributed over 60% of the variance in the ToM scores of 119 pairs of 42-month-old twins to genetic factors. But a much larger follow-up study on 1,116 pairs of 60-month-old twins found genetics to account for only 7% of the variance in false-belief test scores [35]. Hughes suggests the discrepancy is likely due to the difference in power between the two studies, or real developmental differences due to the different ages of the two sets of twins.

In genetic studies it is essential that the phenotype under study is delineated appropriately and this can be especially challenging for psychological genetics. The ability to link a psychological trait measure to specific areas of the brain, while not essential, would engender a greater degree of optimism that a more specific model of heritability might be determined than that using a trait which cannot be linked to any kind of biological marker – and the more specific the biological marker, the better. Anokhin et al. [36] analyzed two event-related potentials (ERPs) of 12-year-old twins responding to happy, fearful and neutral facial expressions and found that genetic factors accounted for 36–64% and 42–62% of individual variation in response to all types of expression. The two ERPs also differed significantly in profile depending on expression. This suggests that these particular ERPs may be biological markers for ToM as measured by the ability to read facial expressions. The possibility of an identifiable genetic influence on these kinds of responses is supported by the finding that the response to happy faces varies according to which allele of the cannabinoid receptor gene (CNR1) individuals carry [37].

The extent to which it is possible to measure a genetic influence on ToM is likely to be affected by the growing recognition of parent-of-origin effect. There is evidence that imprinted genes may influence ToM. Imprinted genes are differentially expressed depending on their parent-of-origin. Differences in levels of expression are controlled without altering the DNA sequence itself, i.e. by epigenetic changes such as gene methylation. Imprinted genes are known to influence mammalian brain development [38] [39] and are implicated in many psychopathologies including some with ToM deficits such as ASC: evidence for a primary role for imprinted genes in the evolution and aetiology of ASC and schizophrenia as diametrically opposed conditions is comprehensively reviewed (and critiqued by open commentary) by Badcock and Crespi [40] and the genomic evidence reviewed by Crespi et al. [41]. The discovery that epigenetic mechanisms influence human gene expression has radically altered our understanding of genetics. For example, it implies opportunities for gene-environment interaction which would otherwise be considered impossible. It raises the possibility that environmental triggers within the lifetime of the individual could alter the *effective* genotype via epigenetic changes, such as DNA methylation [42] [43] [44] [45].

Domes et al. [46] found male Eyes scores were improved when participants were given oxytocin and Rodrigues et al. [47] found Eyes scores were associated with variations in the oxytocin receptor gene (OXTR). Furthermore, Gregory et al. [48] found an association between methylation status of OXTR and ASC. These findings provide one possible mechanism by which imprinting status, i.e. gene methylation, might influence ToM.

It is well known that boys are more often diagnosed with ASC than girls, the ratio being at least 4 to 1. However, as yet, attempts to clearly identify an X-linked gene or sex-specific maternal effect that might explain this sex difference in ASC have failed. The X-chromosome is known to carry a relatively high number of genes effecting cognition and the presence of a single X chromosome in males means that measures of associated traits may show greater variance in males than females while mean scores would be the same in both sexes [49]. The greater variance in a cognitive trait could explain why there tend to be more males than females with extreme trait scores but it cannot account for differences in mean scores. Imprinted X-linked genes, however, alter both the variance and the mean, explaining both sex-differences in susceptibility to extreme trait scores and general sex differences in mean scores.

Females with Turner's Syndrome have one X chromosome (either maternal, X_m_, or paternal, X_p_) and no Y chromosome, and consequently express only the maternal or paternal X-linked genes. This provides a natural human corollary to the ‘knock-outs’ commonly used in animal genetics. Furthermore, the behavioural phenotype of Turner's Syndrome overlaps with that of ASC. Both individuals with ASC and those with Turner's Syndrome fail to recognize facial expressions of fear or ascertain gaze direction, both components of ToM [4] [5]. The impairment in interpreting eye gaze seen in Turner's Syndrome individuals implies that having two X chromosomes may afford some protection against this kind of deficit [50]. Social cognition has been found to be worse in Turner's Syndrome individuals having a maternally derived X chromosome [51]. Brain imaging studies have found evidence of abnormally increased superior temporal gyrus volumes in X_m_ Turner's Syndrome females compared to X_p_ Turner's Syndrome females and controls [52], an area of the brain shown to be selectively activated while interpreting emotion from eye contact [53]. This suggests that there is a parent-of-origin effect on the neural substrate for ToM which is associated with an imprinted, X-linked gene and that the ToM deficit typical of ASC might be associated with a maternal X-linked gene.

Some specific X-linked genes influencing the recognition and processing of facial expressions have been identified. A 5 Mb region of Xp11.3–4 has been associated with facial fear recognition: in particular, the quantitative trait locus EFHC2 has been found to account for over 13% of the variance in fear recognition [54]. X-linked genes(s) influence the function of the pathway that begins at the retina following direct eye contact and produces an emotional response via the amygdala [55]. However, Mazzola et al. [56] did not find a difference in facial fear recognition depending on whether the single X chromosome was maternal or paternal in origin.

Imprinted genes might also affect ToM via executive function. The general gross development of the murine prefrontal cortex is associated with preferential maternal gene expression [38] and there is evidence for preferential maternal gene expression affecting aspects of executive function in mice [57] [58] and humans [59].

### Environmental influences on ToM: the family

Environmental influences which have been associated with increasing ToM skills generally operate via the family or peer group. A landmark study by Perner et al. [60] found that a child's ToM skills increased with the number of siblings, a finding replicated by Jenkins and Astington [61]. Later studies, however, have varied considerably. Peterson and McAlister found that only child-aged sibs had this influence and not infants or adults. Ruffman et al. [62] found that only older sibs increased ToM skills and more recently Farhadian et al. [63] found that birth order influenced ToM skills in preschoolers. Sibling sex may also be a factor as Ruffman et al. [62] found opposite sex pairs to have better ToM skills than same sex pairs. Other studies, however, found no relationship between age or number of siblings and ToM skills [64] [65]: Lewis et al. [66] found that ToM skills increased with the number of other adults and non-siblings interacting with a child, i.e. the total network of interactions has to be taken into account. It may also be that interacting with minds more different to the child's own increases ToM which is why Wright, Cassidy et al. [67] found twins to have relatively poor ToM skills and this may also be why opposite sex siblings have better ToM skills.

Parental attitudes and behaviours influence ToM skills: in practice, studies tend to focus heavily on maternal influences and there is very little, if any, data from fathers. Maternal talk about mental states is associated with increasing ToM skills [68] and the quality of that maternal talk can have further impact, e.g. explanations of mental states [69] [70] and mind-mindedness [71].

While the details are complex and still subject to investigation, the overall evidence for the direct influence of maternal behaviour and interaction on the development of the child's ToM skills is indisputable. However, there is also evidence implying a genetic component and, furthermore, that some of that component may show parent-of-origin effects – very likely maternal. This makes differentiating between environmental and genetic effects especially demanding. The experimental design outlined below uses a dataset comprising scores from full and half-siblings that can potentially differentiate between genetic and environmental influences using not only classical Mendelian models of heritability but also models assuming parent-of-origin effects. Structural equation modelling is commonly used in the analysis of twin studies and has been adapted here for use with full and half-siblings. The limiting factors are the size of the smallest informative sibling category and the balance of siblings raised together compared to those raised apart. Consequently, although a maternal influence was identified, it was not possible to differentiate between genetic and environmental influences in this particular sample although the experimental design has the potential to do so.

## Methods

### Ethics statement

This project has ethical approval from the Cambridge Psychology Research Ethics Committee (Application No: 2005.07). Consent was obtained from all participants. Where the data was collected online, this was part of the registration process. Where the data was collected by paper and post, there was a written consent form.

### Participants

The dataset comprised 70 pairs of full siblings (38 pairs of sisters and 32 pairs of brothers), 25 pairs of maternal siblings (9 pairs of sisters, 4 pairs of brothers and 12 brother-sister pairs) and 15 pairs of paternal siblings (9 pairs of sisters and 6 brother-sister pairs). Siblings were recruited by advertising to the student and general population. Half-siblings were relatively difficult to recruit as were males compared to females. There were no significant differences between the mean Eyes scores from the student and general population. The mean age was 29 (sd = 13).

### Measures

As well as Eyes, participants also completed the Empathy Quotient (EQ), Autism Quotient (AQ) and a Behavioural inhibition inventory (BIS) [72] [73] [74] as part of a larger study. The EQ and AQ were developed to measure ASC traits in the general population. The AQ consists of five 10-item subscales: Social Skills, Communication, Imagination, Attention Switching and Attention to Detail. The EQ, AQ and BIS are all self-report questionnaires, comprising 40, 50 and 6 forced-choice four-point Likert scale items respectively. The EQ items are scored 0, 1, or 2: AQ items are scored 0 or 1. The BIS inventory was developed to measure aspects of behavioural inhibition and comprises six forced-choice four-point Likert scale items scored 1, 2, 3 or 4. All tasks were administered by paper and post.

### Statistics

Statistical tests were carried out using SPSS 16.0 (SPSS Inc., Chicago, IL) for Windows. Structural equation modelling was carried out using MPlus 5 (Muthén and Muthén, Los Angeles, CA).

## Results and Discussion

### Testing Eyes scores for differential parental influences

In an attempt to differentiate between parental influences due to imprinted genes and environmental influences on Eyes, correlations between the Eyes scores of pairs of full- and half-siblings were compared according to how much genotype they share with respect to both the X chromosome and autosomes from each parent (Table 1). The inclusion of half-siblings enables the influence of a shared mother or a shared father to be compared.

**Table 1:** Mean percentage DNA shared by full and half-siblings by chromosome type.

Sibs	Paternal X	Maternal X	Paternal Autosomes	Maternal autosomes
Full sisters	100	50	50	50
Full brothers	0	50	50	50
Full bro-sis	0	50	50	50
Paternal sisters	100	0	50	0
Paternal brothers	0	0	50	0
Paternal bro-sis	0	0	50	0
Maternal sisters	0	50	0	50
Maternal brothers	0	50	0	50
Maternal bro-sis	0	50	0	50

If Eyes scores are influenced by imprinting, then the actual correlations between pairs of siblings will differ from those expected following classical Mendelian heritability in a predictable way, depending on what kind of imprinting and/or sex-linkage is influencing the trait (Table 2). For example, in Mendelian heritability, the expected correlation between full siblings would be twice that of half siblings, but if there is preferred maternal expression of the gene influencing the trait, then maternal siblings will have the same correlation as full siblings, and the group of full and maternal siblings will have twice the correlation of paternal siblings. Non-imprinted X linkage was not included in this analysis. Although it is theoretically possible to distinguish between imprinted and non-imprinted X-linkage, this can be more easily analyzed by conventional means (such as classical pedigree or linkage analysis). The hypothesis to be tested is therefore that a model indicating preferential maternal or paternal influences on Eyes scores will show a different fit to the data compared to one assuming Mendelian heritability.

**Table 2:** Predicted relative order of correlations between siblings by model of preferential gene expression.

Sibs	Model of gene expression assumed					
	Mendelian	Maternal autosomal	Paternal autosomal	Maternal X*	Paternal X	Paternal Xr*
Full sisters	2	2	2	2	4	3
Full brothers	2	2	2	3	0–2	2
Full bro-sis	2	2	2	2	0	1
Paternal sisters	1	0	2	0–1	4	2–3
Paternal brothers	1	0	2	0	0	0
Paternal bro-sis	1	0	2	0	0	0
Maternal sisters	1	2	0	2	0	1
Maternal brothers	1	2	0	3	0–2	2
Maternal bro-sis	1	2	0	2	0	1

Higher numbers denote stronger correlation.

*Assuming random inactivation of one X in females. Maternal X expression without random inactivation is indistinguishable from Maternal autosomal expression. In the case of preferential paternal X expression without random inactivation, values for full and maternal brothers reflects the possibility of zero to full maternal X expression in the absence of a paternal X.

In order to partition parent-of-origin effects into genetic and environmental components, whether siblings are raised together or apart, is an important aspect of the experimental design. In practice, it proved extremely difficult to recruit participants in a balanced way; in particular, most of the maternal siblings were raised together and most of the paternal siblings were raised apart (all the full siblings were raised together). This was refected in the parameters of the path analysis and the implications are discussed below.

Eyes scores were negatively correlated with age (in both sexes) but the relationship is not significant at this sample size and scores were not age-adjusted (*r* = −0.114, *p* = 0.074). The difference in scores between siblings was not related to their difference in age (*r* = 0.033, *p* = 0.727). As in previous studies males scored lower than females (male mean 26.47 sd 3.8741 and female mean 27.301 sd 3.829) but the difference was not significant in this sample (*t* = −1.670 (221), *p* = 0.096).

Table 3 gives the sibling correlations for Eyes corresponding to the models of genomic imprinting derived from Table 2. There are six low-scoring outliers where Eyes <18: two pairs of full sisters, one pair of full brothers, one full brother-sister pair, one pair of maternal brothers and one maternal brother-sister pair. Sibling correlations are also given after removing these low scores (Eyes Adj).

**Table 3:** Pearson correlations (*r*) between siblings for Eyes scores and Eyes scores adjusted by removing the low-scoring outliers (Eyes Adj >17).

			Eyes		Eyes Adj
Model	Category	n	*r*	*n*	*r*
Mendelian	fsib	70	0.177	67	0.195
	PM	40	0.234	37	0.146
Maternal autosomal	FM	95	**0.272** **	90	**0.245** *
	psib	15	**−0.175**	14	**−0.255**
Paternal autosomal	FP	85	0.096	81	0.104
	msib	25	0.417*	23	0.319
Maternal X	FMB	36	**0.394** *	34	**0.338** '
	Else	59	**0.090**	55	**0.085**
Paternal X	FPS	47	0.047	44	0.055
	Else	63	0.318*	60	0.250'

**Correlation is significant at the 0.01 level (2-tailed).

*Correlation is significant at the 0.05 level (2-tailed).

'Correlation is significant at the 0.1 level (2-tailed).

For each model, the two categories of sibling pairs are derived from Table 2. In each case, a possible fit (in bold) is indicated by the second correlation being less than the first.

The contrast between *r* (maternal sibs) = 0.417 and *r* (paternal sibs) = −0.175 is striking (for Eyes the difference has *p* = 0.136 and for Eyes Adj *p* = 0.096). Although 15/25 of the pairs of maternal sibs were living at the same address, *r* (maternal sibs at the same address) = 0.495 while *r* (maternal sibs at different addresses) = 0.467; i.e. they are virtually the same. To be absolutely sure that siblings were not conferring over each other's answers, the group of maternal sibs at the same addresses were analyzed to see if their incorrect answers were incorrect in the same way. This does not appear to have been the case since the incorrect answers matched in only 26/110 cases: 10 of these occurred in one sib pair who scored particularly badly and it might be that one or both sibs struggled so much with the test they did confer on some answers albeit somewhat unsuccessfully.

Given the well-documented influence of the family on ToM development, Eyes scores of participants were analyzed for differences depending on whether they were full- or half-siblings, i.e. to detect the influence of family structure. No significant differences were found in general or by sex.

**Figure 1:**
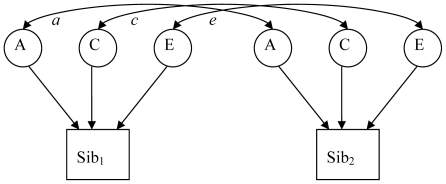
Path diagram (above) and parameter values (below) for model fitting. *a* = covariance in additive genetics and *c* = covariance in shared environment between siblings. Values for *a* represent the strongest form of the model assumed.

Having identified possible models for testing from Table 3, the goodness of fit was tested by structural equation modelling using MPlus. From Table 3, the best fitting models are for preferential maternal autosomal and maternal X-linked expression. Figure 1 shows the path diagram for the model fitting and the parameter values for the genetic and shared environment components. The shared environment parameters were adjusted to reflect the proportion of siblings raised together vs raised apart. Table 4 gives the χ^2^ goodness of fit of the Mendelian and best fitting models from Table 3. The adjusted models without the outliers are also given in Table 4. The genetic models used assume complete imprinting as though the gene(s) influencing the trait were completely, rather than partially imprinted. This is a conservative approach where detecting parent-of-origin effects will depend on the effect size of any imprinted genes being sufficient to shift the expected correlations between siblings away from those expected due to Mendelian gene expression: very small effects will not be detected. As such it is more dependent on sample size than a conventional twin study.

**Table 4:** Model-fitting statistics for Eyes and Eyes Adj testing Mendelian, maternal and X-linked maternal models of heritability.

		Eyes			Eyes Adj		
Model		ACE	AE	CE	ACE	AE	CE
**Mend**	**χ^2^ (df)**	16.7 (6)	16.9 (7)	16.7 (7)	6.55 (6)	6.56 (7)	6.55 (7)
	***p***	0.01	0.02	0.02	0.36	0.48	0.48
**Am**	**χ^2^ (df)**	13.1 (6)	13.1 (7)	13.3 (7)	2.72 (6)	**2.90 (7)**	**2.96 (7)**
	***p***	0.04	0.07	0.07	0.84	**0.89**	**0.89**
**Xm**	**χ^2^ (df)**	9.32 (6)	9.99 (7)	9.99 (7)	3.02 (6)	**3.55 (7)**	5.30 (7)
	***p***	0.16	0.19	0.19	0.81	**0.83**	0.63

Abbreviations: A, genetic influences; C, shared environmental influences; E, non-shared environmental influences.

The best fitting models are in bold.

From Table 4, the best-fitting model for Eyes (i.e. where **χ^2^** is *least* significant) is for a maternal effect that is stronger on males than females. The two most difficult categories of siblings to recruit were maternal siblings raised apart, and even more difficult, paternal siblings raised together. This led to a pattern of shared environment parameters (*c* values in Figure 1) making it impossible to distinguish between a maternal effect due to maternally expressed genes vs one that acts via shared maternal environment. In Table 4 this can be seen in the closeness of the **χ^2^** values for CE (with shared, C, and non-shared, E, environmental influences) and AE (with genetic, A, and non-shared environmental, E, influences) models: they cannot be distinguished. In particular, since females were more easily recruited than males and maternal siblings more easily than paternal, there were no pairs of paternal brothers in this sample. A larger study able to recruit a sample of paternal brothers would be able to distinguish between preferential maternal X-linked gene expression and a maternal effect acting via the environment – or establish a combination of the two.

Removing the low-scoring outliers greatly improves the fit of all the models with the best-fitting models being for a maternal effect, on both sexes and also specifically on males. The increase in **χ^2^** for the CE model vs the AE model for maternal-X linked expression could imply that genetics explains more of this influence than shared environment.

The difference in the model fitting between Eyes and Eyes Adj suggests that the influence on the low scores may be different to the influences on the scores over the normal range. Low scorers in the range 14–17 had siblings in the range 21–31.

### What is the Eyes test actually measuring?

The Cronbach's alpha for Eyes was 0.481, well below the acceptability threshold of 0.7. This reflects the very low inter-item correlations, most of which were below 0.2, with many being negative. This implies that there is very little, if any, relationship between the ability to interpret any one expression and any other expression. There were no particular associations between items falling into theoretical subgroups such as expressions of dominance, submission, anxiety, positive affect, negative affect or friendliness. There was no thematic relationship between those items which are most often answered incorrectly and those answered correctly (Table 5). Apart from reading facial expressions, interpreting the movement and direction of eye gaze with respect to the interest and intention of the subject is also associated with ToM and empathy [75]. However, the ability to answer correctly did not depend on whether the eyes were directed toward or away from the viewer or the sex of the model.

**Table 5:** Correct responses to Eyes items in quartiles depending on how often they are correctly identified.

1 Least correct	2	3	4 Most correct
Uneasy	Insisting	Upset	Playful
Cautious	Worried	Desire	Fantasizing
Doubtful	Anticipating	Despondent	Pre-occupied
Decisive	Fantasizing	Regretful	Accusing
Tentative	Hostile	Sceptical	Friendly
Defiant	Cautious	Contemplative	Pre-occupied
Interested	Interested	Thoughtful	Flirtatious
Reflective	Concerned	Pensive	Nervous
Confident	Distrustful	Serious	Suspicious

The lack of correlation between the ability to identify items correctly, even those illustrating the same mental states, raises questions concerning what underlying latent trait Eyes might actually be measuring. Table 5 shows that similar expressions range considerably with regard to successful interpretation. At least eight of the items are essentially differentiating ‘pre-occupied’ by using various other terms such as ‘pensive’ and ‘thoughtful’. Many of the other terms overlap considerably such as ‘cautious’ and ‘suspicious’. One might expect participants that successfully identify one term to be successful with similar terms but this is not generally the case. Perhaps the particular contrasting terms offered as alternatives are more important in determining success, i.e. what is being tested is the ability to distinguish what the expression is *not* rather than any certainty about what it *is*. For example, there are two very similar items, 9 and 22, where the correct response is ‘pre-occupied’. In a principal components analysis of Eyes (producing 15 components with Eigen values>1) item 9 is one of 12 items in PC1 accounting for 7.2% of the variance in Eyes scores, while item 22 is the sole item comprising PC5, accounting for 4.2% of the variance. A previous study [76] found that accuracy decreased as the number of positive (as opposed to neutral or negative) distractors increased.

It is not clear what advantage there really is in using so many different terms. If what is being tested is the ability to read expressions rather than understand and apply language then it should not matter if the same relatively common terms are used repeatedly. Carroll and Young [77] administered Eyes, EQ and a test of vocabulary, the Wechler Abbreviated Scale of Intelligence Vocabulary subtest (WASI-V), to 48 University students. WASI-V scores showed a small positive correlation with Eyes: *r* = 0.092 and the interaction between EQ and Eyes was increased (from *r* = 0.364 to *r* = 0.406) by controlling for WASI-V scores. In this study, the interaction between EQ scores and Eyes for the larger sample of 262 was considerably less than Carroll and Young's study: *r* = 0.185 *p* = 0.011 but similar to Voracek and Dressler [78]: *r* = 0.2, *p*<0.001, *n* = 423.

To further investigate the traits associated with Eyes scores, they were entered into principal components analyses with the BIS, EQ and AQ subscale scores to assess how a reduction of the data would group the scores into components. BIS, as a measure of behavioural inhibition, is a proxy for executive function, together with the two attention-oriented AQ subscales, Attention Switching and Attention to Detail. A higher order factor analysis places the first four subscales in one higher order factor termed ‘Social Interaction’ with Attention to Detail as a second higher order factor [79]. The AQ overall has an inverse relationship with EQ scores, with high scores being associated with weak empathy.

The principal components analysis was first performed for the group of first siblings (Sib 1) and second siblings of a pair (Sib 2) separately in order to eliminate confounding issues of relatedness and the results being the same, the two groups were then combined, again giving the same results. Those results are summarized in Table 6. The AQ subscales were retained as using the total AQ scores, or grouping the four socially-oriented subscales reduced the Kaiser-Meyer-Olkin Measure of Sampling Adequacy (KMO) to <0.5. In the final analysis KMO = 0.697 and Bartlett's test of Sphericity was significant (χ^2^ = 253 (28), p<0.001).

**Table 6:** Principal components 1 and 2 (loadings >0.4) from the principal components analyses of the Eyes, BIS, EQ and AQ subscale scores.

Score	PC1	PC2
Eyes		0.526
Behavioural Inhibition (BIS)		0.828
Empathy Quotient (EQ)*	−0.738	
Attention Switching	0.643	0.483
Attention to Detail		0.405
Communication	0.868	
Social Skills	0.787	
Imagination	0.509	

*The EQ loading is negative because the subscales AS, C, SS and I are all measured in the opposite sense, i.e. high scores are associated with poor skills and low empathy.

From Table 6, PC1 comprises the socially-oriented subscales of the AQ with EQ scores. The strongest loadings on PC2 are BIS and Eyes, with moderate contributions from both Attention Switching and Attention to Detail. This suggests that variance in Eyes scores is associated with executive function as measured by behavioural inhibition and attention rather than with empathy and social skills. This is in close agreement with Bull et al.'s dual task study using a shortened Eyes task (12) and has bearing on the unresolved question of whether ToM is a trait that can be defined and isolated to some, or any, extent from more general, less specific executive function. Taken together with the lack of inter-correlation in item responses, this could suggest that it is Eyes, rather than ToM, that depends on executive function, i.e. Eyes is not actually measuring ToM. However, as noted above, there is evidence that Eyes does in fact measure ToM: e.g. Sabbagh and Seamans 6 found parents' Eyes scores to be correlated with their children's performance on Wellman and Liu's [80] Children's Theory-of Mind Scale. It may be, then, that Eyes measures an aspect of ToM that is more related to executive function than specific measures of social cognition. A further issue to consider here (but beyond the scope of the present discussion) is the relationship between false belief tests (such as form part of Wellman and Liu's scale) and ToM, which may not be the gold standard measure of ToM it is commonly taken to be [81].

As noted above, the recognition and processing of some specific emotions is associated with specific neural pathways and possibly specific genes and may reflect independent evolutionary histories, reflecting different selective pressures. In which case, any test which includes a range of mental state processing may be unlikely to capture variance in ToM-specific influences and will target the kind of skills which underpin mental state processing as a cognitive process in general rather than one that is associated with mental state processing in particular. Targeting mental state processing itself (if indeed there actually are such relatively independent processes) may require tasks that are specific to particular kinds of mental state processing and the major difficulty with this is developing a task with sufficient variance, given its specificity. Eyes was developed to be challenging enough to give reasonable variance without the ceiling effects in the normal population which are commonly found in simpler tests developed in the assessment of psychiatric conditions.

There are some discrepancies across studies using Eyes which suggest that the psychometric properties of this test require further investigation. The original paper (1) reports Eyes as having a correlation with AQ scores of r = −0.53. In this study, *r* (Eyes×AQ) = −0.079, and in another study using Eyes (61) *r* (Eyes×AQ) in males = −0.13, and in females = −0.017. These values are all significantly different from the original paper (*p*<0.001) but not significantly different from each other (all *p*>0.3). Eyes was originally developed to measure the deficit in ToM associated with ASC, in which case a strong interaction with AQ scores would be expected. The original study included 14 diagnosed cases of ASC (outliers are not mentioned): there were none in the present study (or 61). It may be that interaction between Eyes and AQ scores is much weaker in the normal population compared to an ASC group and this may be because the aspect of ToM that is primarily associated with the ToM deficit observed in ASC is not entirely continuous with the normal population. This is supported by the finding that removing low Eyes outliers greatly improves model-fitting, i.e. neither Mendelian heritability nor a maternal effect (of any kind) has any explanatory power with regard to the lowest Eyes scores. If this could be confirmed in a larger dataset then it would imply that there is a different influence acting to produce the lowest Eyes scores: the physiological mechanism by which Eyes scores are influenced may or may not be the same but the heritable or maternal influence producing low scores may be qualitatively different.

### Summary

There appears to be a maternal influence on Eyes scores in general and it also appears that brothers' Eyes scores are more similar than sisters'. It is unclear, however, how much of this influence is genetic and how much is environmental, although the experimental design does have the potential to differentiate between genetic, shared and non-shared environmental influences in a larger, more comprehensive sample. Since males tend to have lower Eyes scores than females, this implies that the influence on males is acting to decrease scores relative to females. Since the model-fitting is greatly improved by removing low-scoring outliers, it may be that there are different influences producing very low scores compared to those in the normal range.

Eyes scores were associated with measures of executive function (behavioural inhibition and attention) rather than empathy or social skills. It may be that Eyes is measuring an aspect of ToM that is highly cognitive (rather than affective) and reflects the use of executive function in the analysis of facial expression in general rather than specific components of ToM which may process specific expressions. Further psychometric analyses are required to further clarify the relationship between Eyes and ToM. In general, care should be taken to compare like with like, with regard to the measures used and the sample profile: it cannot be assumed that measures of ToM are equivalent or developmentally stable.

Whether the general maternal influence on Eyes is genetic, environmental or a combination of both, these results suggest it is likely to act via executive function.
